# Repetitive Transcranial Magnetic Stimulation for Mood and Anxiety Symptoms in Complex Clinical Population

**DOI:** 10.1192/j.eurpsy.2026.10712

**Published:** 2026-07-31

**Authors:** A. V. Samokhvalov, A. Doomra

**Affiliations:** 1 Interventional Psychiatry, Homewood Health Centre, Guelph; 2 Department of Psychiatry & Behavioural Neurosciences, McMaster University, Hamilton; 3 ASU/CPC, Homewood Health Centre, Guelph, Canada

## Abstract

**Introduction:**

Repetitive Transcranial Magnetic Stimulation (rTMS) is an effective neuromodulation treatment with proven effectiveness in multiple clinical trials aimed primarily at treatment-resistant depression (TRD) [1, 2]. Its effectiveness in complex clinical populations needs further evaluation. This study examined the effectiveness of rTMS in patients with TRD of mixed etiology and multiple comorbidities.

**Objectives:**

To evaluate the effectiveness and feasibility of rTMS in complex clinical populations.

**Methods:**

Observational study. Quick Inventory of Depressive Symptomatology (QIDS). Generalized Anxiety Disorder questionnaire (GAD-7). Descriptive statistics.

**Results:**

We have treated 151 patients, 86 women (57%) and 65 men (43%), average age 42.9±16.4 years. Vast majority (90.7%) of patients had a primary diagnosis of major depressive disorder, 6.6% - bipolar depression, and four patients had other disorders. The standard scales were used to quantify the severity of depressive symptoms (QIDS) and anxiety (GAD-7). The average baseline scores for depression and anxiety were 17.0±4.9 and 12.9±5.3, respectively. The patients received an average of 22.4±7.5 treatments. Three quarters (74.2%) of patients received the full course of 20-30 treatments as planned. The average end-of-treatment (EoT) scores for severity of depressive symptoms and anxiety were 11.2±6.2 and 8.7±5.7, respectively. The rates of improvement (>25% score reduction) and complete resolution of depressive symptoms were 62.0% and 16.0%, respectively. The rates of improvement and complete resolution of anxiety symptoms were 49.6% and 16.8%, respectively. There was significant difference between the bipolar and major depression subgroups symptomatology changes and minimal differences between the subgroups of patients with depression and with or without comorbidities. Patients with MDD and comorbidities had more pronounced anxiety severity than the rest (see Figure 1).

**Image 1:**
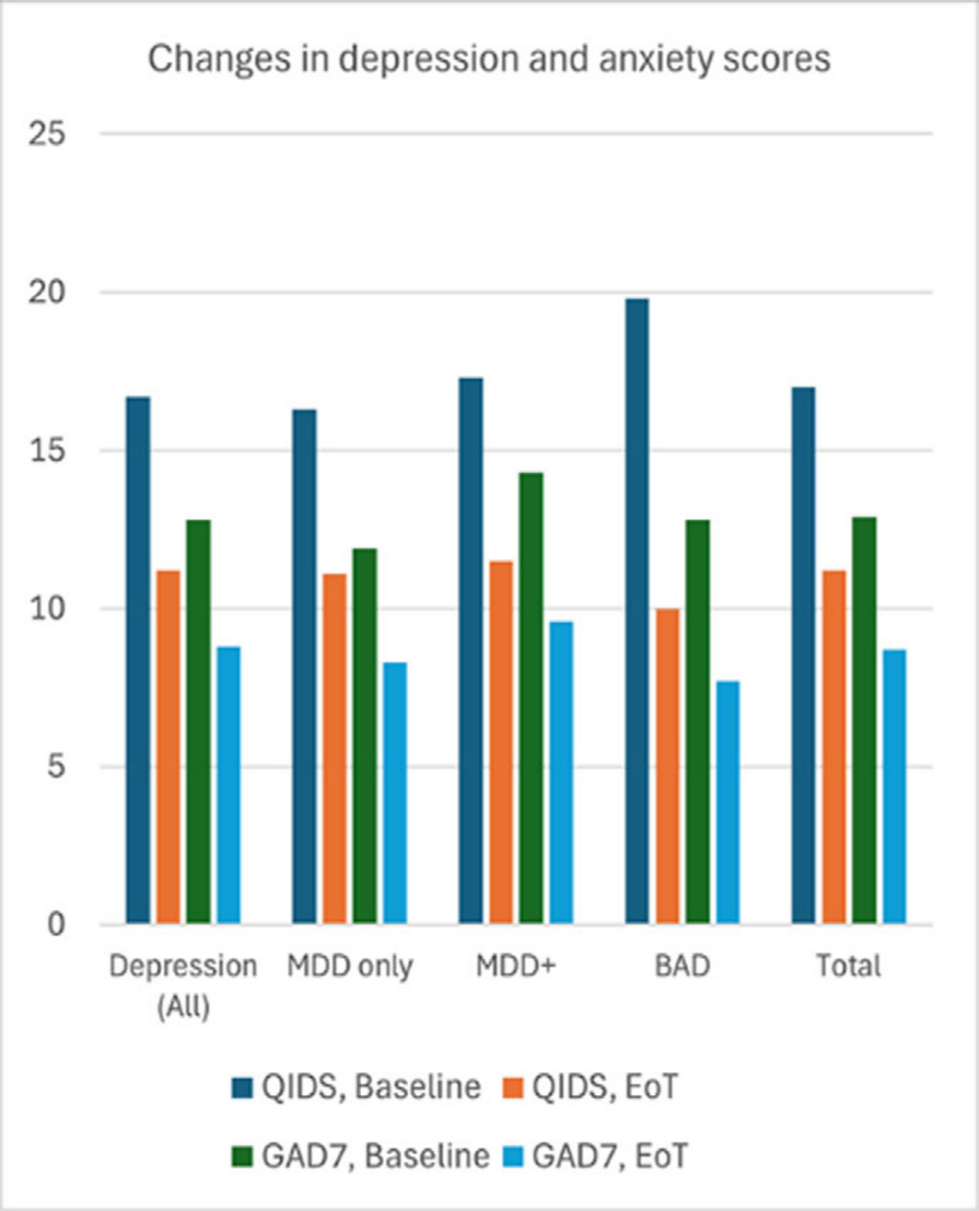
Image 1: Long description.

**Conclusions:**

rTMS provides significant improvement and recovery rates in complex clinical populations and is well tolerated, both in patients with unipolar and bipolar depression and with comorbid conditions. We recommend wider implementation of rTMS for treatment in complex clinical populations.

**Disclosure of Interest:**

None Declared

## Long descriptions

### Image 1: Long description

A vertical bar chart titled Changes in depression and anxiety scores. The y-axis represents scores from 0 to 25 in increments of 5. The x-axis lists five categories. Depression All, M D D only, M D D plus, B A D, and Total. A legend at the bottom identifies four metrics. Q I D S Baseline in dark blue, Q I D S E o T in orange, G A D 7 Baseline in green, and G A D 7 E o T in light blue.

Data trends show a consistent decrease from baseline to E o T for both scales in all groups.

* Depression All. Q I D S drops from approximately 17 to 11. G A D 7 drops from 13 to 9.

* M D D only. Q I D S drops from 16 to 11. G A D 7 drops from 12 to 8.

* M D D plus. Q I D S drops from 17 to 11.5. G A D 7 drops from 14 to 10.

* B A D. Q I D S drops from 20 to 10. G A D 7 drops from 13 to 8.

* Total. Q I D S drops from 17 to 11. G A D 7 drops from 13 to 9.

Navigate back to Image 1:.

